# The Paradox of Akt-mTOR Interactions

**DOI:** 10.3389/fonc.2013.00165

**Published:** 2013-06-20

**Authors:** Lakshmipathi Vadlakonda, Abhinandita Dash, Mukesh Pasupuleti, Kotha Anil Kumar, Pallu Reddanna

**Affiliations:** ^*1*^Department of Zoology, Cell Biology and Enzymology, Kakatiya University, Warangal, India; ^*2*^SRM Research Institute, SRM University, Kattankulathur, India; ^*3*^Department of Animal Sciences, School of Life Sciences, University of Hyderabad, Hyderabad, India; ^*4*^National Institute of Animal Biotechnology, University of Hyderabad Campus, Hyderabad, India

**Keywords:** FoxO, rictor, Akt, insulin/IGF signaling, glucose transport

## Abstract

The serine threonine protein kinase, Akt, is at the central hub of signaling pathways that regulates cell growth, differentiation, and survival. The reciprocal relation that exists between the two activating phosphorylation sites of Akt, T308 and S473, and the two mTOR complexes, C1 and C2, forms the central controlling hub that regulates these cellular functions. In our previous review “PI3Kinase (PI3K)-AKT-mTOR and Wnt signaling pathways in cell cycle” we discussed the reciprocal relation between mTORC1 and C2 complexes in regulating cell metabolism and cell cycle progression in cancer cells. We present in this article, a hypothesis that activation of Akt-T308 phosphorylation in the presence of high ATP:AMP ratio promotes the stability of its phosphorylations and activates mTORC1 and the energy consuming biosynthetic processes. Depletion of energy leads to inactivation of mTORC1, activation of AMPK, FoxO, and promotes constitution of mTORC2 that leads to phosphorylation of Akt S473. Akt can also be activated independent of PI3K; this appears to have an advantage under situations like dietary restrictions, where insulin/insulin growth factor signaling could be a casualty.

## Introduction

Protein kinases have been implicated in affecting many aspects of metabolism and cell fate and play key roles in the pathogenesis of human diseases, including metabolic disorders, degenerative diseases, and cancer. Akt or Protein kinase B was discovered in the year 1991, as a novel serine threonine (ser/thr) protein kinase, by three independent groups. It was variously named as rac protein kinase (rac, for related to the A and C protein kinases; RAC-PK) (Jones et al., 1991); Protein kinase B (between protein kinase A and C) (Coffer and Woodgett, 1991); or Akt (designated after the oncogenic provirus, AKT-8) (Bellacosa et al., 1991). Downward (1995) proposed the use of the kinase name as Akt/PKB to avoid confusion with Ras related GTPase RAC. The use of Akt has become popular in literature since then. Structurally, the protein Akt/PKB can be divided into three regions; the N-terminal pleckstrin homology containing (AH/PH) domain, the centrally located kinase domain, and a C-terminal regulatory domain containing the hydrophobic motif (HM) phosphorylation site (Nicholson and Anderson, 2002). Akt is activated in response to the insulin or insulin growth factor signaling (IIS). Akt exists in three isoforms Akt1, 2, 3 with a varying tissue distribution but with similar domain structure (Jones et al., 1991; Konishi et al., 2007).

## Akt is the Central Hub of Insulin/Insulin Growth Factor Signaling

Insulin or insulin like growth factor signaling is the major signaling pathway that responds to the nutrient signals. Insulin is the major hormone promoting the anabolic activity in the body. Deregulation of this pathway had been shown to be the principal cause of the metabolic syndromes like type 2 diabetes, obesity, and cancer (Laplante and Sabatini, 2012; Boosani and Agrawal, 2013; Zhu et al., 2013). White (2003), in his brief review, presented the summary of events that led to the description of the role of Akt in insulin signaling pathway (see also the pathway map http://stke.sciencemag.org/cgi/cm/CMP_12069?cookietest=yes) (White, 2003). In brief, the IIS pathway is activated by the insulin or insulin like growth factor (IGF) binding to the insulin/IGF receptor, a hetero-tetramer protein, comprising of two α and two β subunits spanning across the membrane. Upon stimulation by IGF, the tyrosine residues of the cytosolic domains are phosphorylated. This leads to the recruitment of the key scaffold protein, the insulin receptor substrate 1 or 2 (IRS 1/2), to the receptor site; IRS in turn activates the PI3Kinase (PI3K)-Akt pathway.

## Phosphorylation of AKT is the Key to its Activation

Akt is phosphorylated at several sites (Mahajan and Mahajan, 2012), although the significance of these phosphorylations remains to be fully understood. Its activity depends on two phosphorylated sites; one located in the catalytic domain [also known as the T- or activation loop, the threonine 308 (Akt T308)] and the second in the HM, the serine 473 (Akt S473). Insulin was shown (Kohn et al., 1995) to activate PI3K mediated activation of Akt. PI3K is a heterodimer consisting of a regulatory p85 subunit and a catalytic p110 subunit, which mediates the phosphorylation of phosphoinositides (PIs) at the three-position of the inositol ring. The enzyme phosphatidylinositol (PI) 3-kinase converts PI 4,5-bisphosphate (PIP2) to the putative second messenger PI 3,4,5-trisphosphate (PIP3) (Fruman et al., 1998). The AH/PH domain was shown to be required for the binding of PIs to PH domain and recruit Akt to the membrane (Franke et al., 1995). There are also reports that Akt can be translocated to the membrane independent of PI3K (Brugge et al., 2007; Mahajan and Mahajan, 2012). Alessi et al. (1996) identified T308 and S473 phosphorylations on Akt as the activating sites, and that these phosphorylations do not depend on one another. A 67 kDa protein was shown to be responsible for phosphorylation of Akt exclusively on T308 and it was purified in 1997 from skeletal muscle by Alessi et al. (1997) and from rat brain by Stokoe et al. (1997), Alessi et al. (1997), and Stokoe et al. (1997). The kinase was named as 3-phosphoinositide-dependent protein kinase 1 [PIPDK; the abbreviation PIPDK is preferred in this article over the original PDK1 (Alessi et al., 1997) to avoid confusion with the pyruvate dehydrogenase kinase, which is also abbreviated as PDK1 in the literature]. An unknown kinase, PDK2, was thought to mediate the S473 phosphorylation. The identity of this unknown kinase was complicated with reports that more than ten heterologous kinases were able to phosphorylate Akt on Ser 473 (reviewed in Dong and Liu, 2005). Among the kinases that are reported to phosphorylate Akt S473, there is a broad consensus, that mTORC2 (mechanistic target of rapamycin complex 2; mTOR was formerly known as the mammalian target of rapamycin) could be the kinase that phosphorylates Akt S473 (Sarbassov et al., 2005). It is reported to phosphorylate Akt under a variety of physiological conditions (Frias et al., 2006). ILK was also shown to phosphorylate Akt S473 in association with rictor and siRNAs against rictor/ILK resulted in inhibition of Akt S473 (McDonald et al., 2008). But, there is no consistency in the reports on ILK’s role in this phosphorylation. Chan and Tsichlis (2001), suggested that ILK could be acting as a scaffold protein. The double-stranded DNA-dependent protein kinase (DNA-PK) has gained some acceptance (Feng et al., 2004; Bozulic et al., 2008) as the possible kinase especially under the conditions of DNA damage (Stronach et al., 2011). An atypical IκB kinase ε and TANK-binding kinase 1 (IKKε/TBK1) was also suggested to phosphorylate S473 in rictor^−*/*−^ cells; it also needed PI3K signaling for activation of Akt (Xie et al., 2011). Tumor necrosis factor α (TNFα) was earlier reported (O’Toole et al., 2001) to promote phosphorylation of Akt exclusively on S473. It is reported that inflammation is associated with hyper active mTORC1 and inhibition of mTORC1 was recently shown to control inflammation (Thiem et al., 2013). Inhibition of mTORC1 and the presence of rictor is required for phosphorylation of AktS473 was earlier reported (Breuleux et al., 2009). It is possible that under the conditions of inhibited state of rictor by mTORC1, activation of IKKε/TBK1 promotes the phosphorylation of Akt S473 for cell survival. TBK1 was recently shown to be an activator of autophagy in the clearance of *Salmonella enterica* (Weidberg and Elazar, 2011).

## Role of Akt S473 Phosphorylation in Cellular Function

There are conflicting views on the role of Akt S473 phosphorylation in cellular function. It has been suggested that phosphorylation of the Ser473 may be independent of its activity (Hill et al., 2001) or it may not be necessary for the full activation of Akt (Moore et al., 2011) and that phosphorylation of Thr308 is a more reliable biomarker than that of Ser473 for Akt activity especially in tumor samples (Guertin et al., 2006; Vincent et al., 2011). There is an increasing evidence that selective mTORC1 inhibition can elicit increased AKT S473 phosphorylation and attenuates the signal effects on tumor cell proliferation (Guertin et al., 2006; Ikenoue et al., 2008; Breuleux et al., 2009). It is clear from the foregone discussion that there is a reciprocal relation between Akt and the two mTOR complexes. IIS mediated phosphorylation of Akt-T308 activates mTORC1, and mTORC2 phosphorylates Akt on S473.

## The Activating Phosphorylations of Akt T308 and Akt S473 have Diverse Downstream Effectors

In most of the phosphoproteomic data on Akt, the two phosphorylation sites are shown to be activated concurrently on IIS signaling and this has led to the suggestion that the phosphorylation at two sites is required for its maximal activity, and IIS stimulates these functions. However, there is a division in the functional role of Akt in cells and the two phosphorylations play critical role in this functional division (Chandrasekher and Sailaja, 2004; Vadlakonda et al., 2013). It has been demonstrated that, Akt-T308 phosphorylated form is essential and S473 is not needed for activation of mTORC1 (Guertin et al., 2006; Rodrik-Outmezguine et al., 2012). mTORC1 activates protein synthesis, S6K and inhibits autophagy; S6K is a feedback inhibitor of IIS and represses the rictor/mTORC2 functions. Actively proliferating cells therefore, require an active mTORC1 to initiate the process of cell cycle but inhibition of mTORC1 and the activation of mTORC2 is required for progression of cell cycle beyond S phase (Vadlakonda et al., 2013). The phosphorylation status of Akt in three different types of leukemia presents an interesting case. Tazzari et al. (2004) reported high levels of S473 phosphorylation in *acute myeloid leukemia blasts* (AML blasts), similarly Nyakern et al. (2006) reported high levels of Akt S473 phosphorylation in mononuclear cells from bone marrow of the patients with high-risk *myelodysplastic syndrome* (MDS) when compared to normal or low risk MDS patients; Gallay et al. (2009) on the other hand, reported higher T308 phosphorylation in patients with AML, which was shown to be associated with high-risk cytogenetics and poor overall survival. Although apparently contradictory, the results reflect the status of proliferation of the cells examined; actively proliferating AML cases have high T308, while the AML blasts and MDS, which are poorly dividing cells have high levels of S473. In our earlier review (Vadlakonda et al., 2013) we suggested that activation of mTORC2, which is the upstream regulator of Akt S473, requires inhibition of mTORC1. mTORC2 has two key functions, phosphorylation of Akt (Sarbassov et al., 2005) and at the plasma membrane mTORC2 was shown to promote reorganization of cytoskeleton by activating RhoA GTPases and protect cell survival by up regulating anti-apoptotic proteins, the BCL2 (Goncharova et al., 2011). It is not clear, how these two functions of mTORC2 are partitioned in actively proliferating cells and quiescent cells. It was shown that mTORC1 activity is needed for translation of mRNA of RhoA GTPases and for mTORC2 mediated cytoskeleton reorganization (Lee et al., 2012). Akt phosphorylated at S473 inhibits FoxO in non-proliferative senescent cells and there appears to be waves of activation and inactivation cycles between Akt, mTOR complexes, autophagy, and FoxO in such cells to keep the survival of the cells intact (Young et al., 2009). Besides, Akt S473 phosphorylation is needed for uptake of glucose (Kumar et al., 2010) and the quiescent or senescent cells require glucose more as they rely on glycolysis for energy needs. It is coming to be realized that in tumor tissue, there is a metabolic symbiosis between non-proliferative cells depending on glucose, while actively proliferating cells on lactate (Semenza, 2008).

## The Presence of Two Phosphorylations in the Phosphoproteomic Data is a Paradox

There is evidence that prior phosphorylation of Akt S473 was required for enabling PIPDK (PDK1) to phosphorylate at T308 (Scheid et al., 2002; Yang et al., 2002). Sabatini group, who demonstrated that mTORC2 is the phosphorylating kinase of Akt S473 also confirmed this (Sarbassov et al., 2005). In our previous review (Vadlakonda et al., 2013), we have shown that Akt S473 phosphorylation follows the inhibition of mTORC1, activation (or reactivation) of FoxO, AMPK, mTORC2, and the progression of cell cycle. This dichotomy in Akt’s relation with mTOR complexes is also reflected in other physiological functions of the cells; for example, anti-apoptotic and proliferative signals from IGF-1 were shown to bifurcate downstream of PI3K in lens epithelial cells (Chandrasekher and Sailaja, 2004). IGF-1-mediated stimulation of the PI3K/p70 S6K cascade was shown to promote cell proliferation, but inactivation of proapoptotic Bad protein and suppression of caspase activation was shown to be independent of PI3K/p70 S6K signaling (Chandrasekher and Sailaja, 2004). A similar situation was also noticed in human non-small cell lung cancer (Vincent et al., 2011). Akt phosphorylated at T308, primarily targets TSC2 and PRAS40 leading to the activation of mTORC1, S6K, and protein synthesis (Kwiatkowski and Manning, 2005), while Akt phosphorylated at S473 was shown to target mainly the FoxO proteins (Guo et al., 2006) and promote anti apoptotic and cell survival pathways (Guan et al., 2011). Akt S473 is a key player in promoting GLUT4 translocation by phosphorylation of As 160 (Zong et al., 2009; Kumar et al., 2010).

## How do the Two Phosphorylated Sites of Akt Translate their Messages to the Diverse Targets?

In our previous review, we suggested that IIS activated PI3K-Akt-mTORC1 is in fact a negative regulator of mTORC2 and FoxO; the two complexes, mTORC1 and mTORC2 regulate each other by a feedback control (Vadlakonda et al., 2013). Such antagonistic relation between mTORC1 and mTORC2 was also recognized earlier (Breuleux et al., 2009). This raises a question whether Akt T308 and Akt S473 also regulate each other? In a perspective article “PDK2: a complex tail in one Akt,” Chan and Tsichlis (2001) presented evidence that in embryonic stem cells which carry inactive PDK1 (PIPDK), phosphorylation of T308 has an inhibitory effect on AktS473 phosphorylation. In a study on pancreatic islet β-cell specific Rictor and phosphatase and tensin homolog deleted on chromosome 10 (PTEN) knockout mice (Gu et al., 2011) demonstrated that βPtenKO mice exhibit a 12-fold increase in AKT-T308 phosphorylation and increase in cell proliferation; rictor null mice on the other hand, were shown to exhibit reduction in β-cell mass, mild hyperglycemia, and glucose intolerance Gu et al. (2011). The authors suggested a critical role for Akt S473 in maintaining the normal β-cell mass and negatively regulates the T308 phosphorylated functions. The two phosphorylations of Akt thus have an antagonistic effects on each other, but in their review Chan and Tsichlis (2001) suggested that the interaction of some unknown molecules in the Akt activation complex might alter its conformation to protect and stabilize the phosphorylations of T308 and S473 on Akt.

## Inter Domain Interactions are Key in Regulation of Akt Functions

As already indicated, Akt contains three main domains; an N-terminal pleckstrin homology containing (AH/PH) domain, the centrally located kinase domain, and a C-terminal regulatory domain containing the HM phosphorylation site. The presence of two linker peptides, one between the PH and catalytic domain, and the other between the catalytic and the c-terminal HM, which could not be crystallized is a major hindrance in obtaining the crystal structure of full length Akt protein (Calleja et al., 2009a), and in studying its inter domain interactions. However, using combined techniques like protein mass spectrometry, Förster resonance energy transfer (FRET) by fluorescence life time imaging microscopy, molecular dynamics, and classical biochemical approaches (Calleja et al., 2009a,b) proposed that in an inactive state the PH domain assumes a PH-in conformation and its interaction with the catalytic domain creates a cavity, which leads to the dephosphorylation of the two activating phosphorylations T308 and S473. Two amino acid residues, tryptophan 80 (W80) in the PH domain, and phenylalanine 469 (F469) in the hydrophobic domain interact to keep this inactive state. Upon stimulation by growth factors, the PH domain assumes the PH-out conformation and translocation of Akt to plasma membrane induces conformational changes in the protein facilitating its phosphorylation of the two activating sites T308 and S473 (Macreadie et al., 1991). Upon phosphorylation, Akt is detached from the membrane and translocates to the target sites in the cytoplasm and nucleus. In an earlier study on activation and inactivation dynamics of Akt signaling, Kunkel et al. (2005) employed biosensors and the real time imaging with FRET, and reported a turnover of the activated Akt with inactive forms at membrane site and that phosphatases promote the inactivation of Akt signaling. The inactivation was shown to be rapid in cytosol when compared to that of the membrane site or in nucleus.

## Phosphatases Regulate the Amplitude and Duration of Kinase Activity of Akt

The PTEN, a lipid phosphatase and a tumor suppressor (Maehama and Dixon, 1998), is one of the key regulators of the PI3K-Akt pathway. It dephosphorylates the D3 position of phosphatidylinositol (3,4,5)-trisphosphate (PIP3). Mutations resulting in the functional loss of this phosphatase leading to up regulation of Akt activity are reported in several cancers (Hollander et al., 2011; Mester and Eng, 2013). Apart from this, Akt activity itself is shown to be controlled directly by three phosphatases, the protein phosphatase 2A (PP2A) (Resjo et al., 2002) and protein phosphatase 1 (PP1) (Xu et al., 2003; Thayyullathil et al., 2011), and PH domain leucine-rich repeat protein phosphatase (PHLPP), which is insensitive to okadaic acid and specifically dephosphorylates the S473 (Bayascas and Alessi, 2005; Gao et al., 2005). An increased phosphorylation of T308 was shown to be associated with reduced PP2A in AML patients (Gallay et al., 2009). These phosphatases also play crucial role in conferring resistance to radiation and chemo therapies; Eke et al. (2010) recently demonstrated that an adapter protein, downstream of focal adhesion, PINCH1 [5 Lin-1, Isl-1, Mec-3 (LIM) domain – containing particularly interesting new cysteine-histidine-rich 1], inhibits protein phosphatase 1α and confers resistance to cancer cells against ionizing radiation and chemo therapies by increasing the Akt phosphorylation.

## The Adenylates ATP/ADP Modulate the Stability of Akt Phosphorylations and Deny Access of Activated Akt to Phosphatase Action

Chan and Tsichlis (2001), in their review, predicted that some unknown molecules in the Akt activation complex might stabilize its phosphorylations. In a recent study, two different groups (Chan et al., 2011; Lin et al., 2012) demonstrated that ATP bound to the phosphorylated Akt protects the phosphorylated sites by preventing their access to the phosphatases, which in turn results in increased phosphorylations of these sites. Adenylate nucleotides, ATP and ADP, were shown to act as “on – off switch” to maintain the stability of the phosphorylations at T308 and S473. Both ATP and the ATP competitive inhibitors were shown to stabilize the two phosphorylation sites, while hydrolysis of ATP to ADP was shown to destabilize the kinase and expose the two phosphorylated sites to phosphatases. Reviewing these two works, Humphrey and James (2012) suggested that Akt creates a cage in the ATP bound form between the two phosphorylations in preventing their access to phosphatases and the stabilized form was shown to detach from the membrane to be transported to the locations of its targets. The phosphorylation at T308 was suggested to increase the affinity between the PH domain and the phosphorylated kinase domain leading to the detachment of Akt from the membrane (Ananthanarayanan et al., 2007). Although the question as to how this stabilization of phosphorylation sites results in the diversified functions of the Akt remains unanswered, it is not difficult to speculate that the energy charge and the localization site of its targets appears to play a critical role in its effects on the downstream targets.

## Energy Charge (ATP/AMP Ratio) of Cells Becomes Crucial in Modulating Akt’s Function

**Figure 1:**
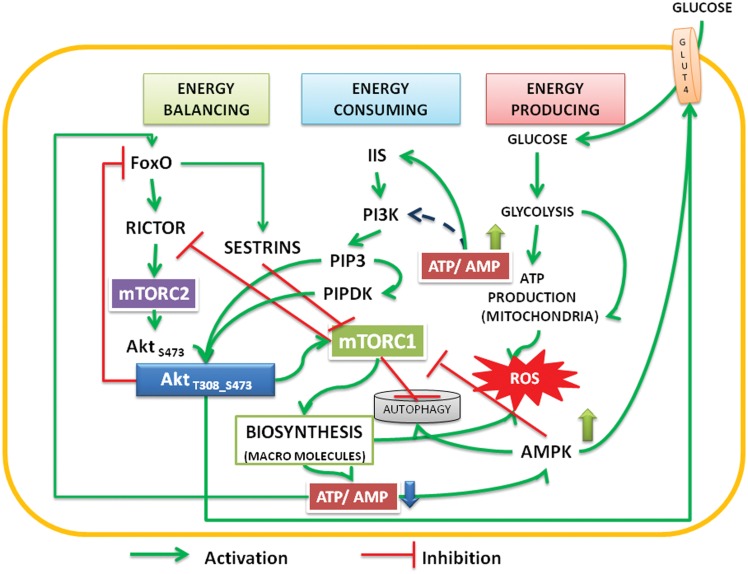
**The interactions and feedback loops between Akt and mTOR complexes and energy consuming and energy producing processes**. Insulin/insulin growth factor signaling (IIS) activates PI3Kinase, which phosphorylates PIP2 to PIP3. Binding of PIP3 to AH/PH domain translocates Akt to the membrane. FoxO transcription factors transcribe rictor leading to assembly of mTORC2 complex, which phosphorylates Akt S473. Activated by PIP3, the PIPDK (originally the PDK1) phosphorylates Akt T308. PI3K-Akt pathway can also be activated independent of IIS (see Mahajan and Mahajan, 2012). The phosphorylations are stabilized by high ATP:AMP ratio, Akt migrates into cytoplasm and nucleus. In the nucleus it phosphorylates FoxO, translocates it into the cytoplasm, and under low energy levels AMPK promotes its translocation into mitochondria. In cooperation with Sirtuin3, FoxO promotes mitochondrial OXPHOS (Peserico et al., 2013). Sestrins, transcribed by FoxO inhibit mTORC1 and the energy consuming processes come down. This up regulates ATP:AMP ratio. At basal level of activation, Akt inhibits AS 160 and activates translocation of glucose transporter (GLUT4) to the membrane there by facilitating the glucose entry into the cells. Activated AMPK also promotes glucose transport under low energy conditions (Schwenk et al., 2010; Wu et al., 2013). The cycle continues under healthy environments. Under inflammatory environment, either due to the Dietary surplus environment or under stress activated conditions, the IIS is destabilized and causes insulin resistance (see Figure 2).

The stabilized phosphorylations under ATP/energy rich environment by activating Akt (Figure 1) primarily target TSC2 and PRAS 40 in cytosol and activate mTORC1 functions. With the reduction in ATP levels under energy consuming processes activated by mTORC1, ADP accumulates, and destabilizes the phosphorylations of Akt; this exposes the phosphorylated sites to phosphatases. In our previous article (Vadlakonda et al., 2013), we proposed that the constitution of active mTORC2 takes place only when FoxO, AMPK, and autophagy are activated and mTORC1 is inhibited. It is well recognized that activation of AMPK and autophagy and inhibition of mTORC1take place only when the energy levels in cells drop (Hardie and Hawley, 2001; Hardie, 2011). The ATP-ADP switch controlling the stability of Akt therefore, depends on the energy charge, a concept defined by Atkinson in 1960s (Ramaiah et al., 1964; Atkinson, 1968). The ratio of concentration of AMP:ATP which varies at equilibrium as the square of the ADP:ATP ratio is maintained by the enzyme adenylate kinase, which is highly expressed in all eukaryotic cells. The role of AMPK in responding to the equilibrium of adenylate pool and the equations related to these interactions were discussed in detail by Hardie and Hawley (2001) and reviewed recently by Oakhill et al. (2011, 2012). An energy charge of healthy cells is maintained around 0.9 (10:1) (Hardie and Hawley, 2001); this was shown to adjust the partitioning of substrates among competing metabolic functions of energy producing or energy consuming processes by balancing the feedback regulation cycles (Hardie and Hawley, 2001). Drugs activating AMPK were shown to dephosphorylate and inactivate Akt but activate the Akt target GSK3 β (King et al., 2006). This confirms that under high ATP:AMP ratio, the targets of Akt will be TSC2 and PRAS 40, whose inhibition activates mTORC1. But under low ATP:AMP ratio, the targets will be the factors that mobilize resources (glucose) for energy production. It is therefore not illogical to speculate that the energy dynamics play critical role in activation inactivation cycles of Akt and modulate glucose up take.

**Figure 2:**
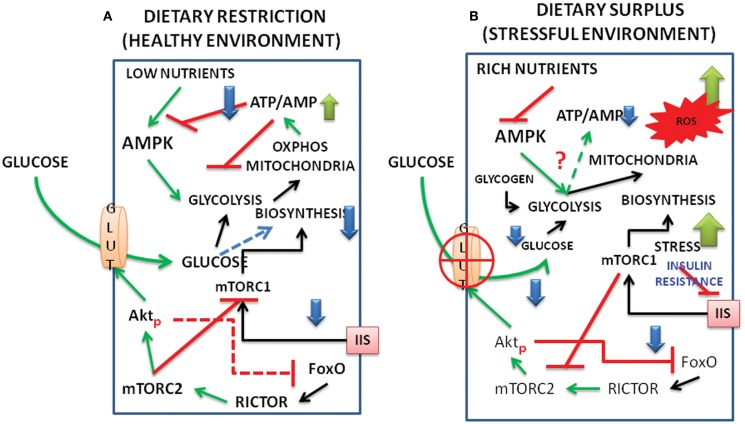
**Schematic representation of interacting signal pathways under (A) dietary restriction and healthy environment and (B) under dietary surplus and stress environment**. The interactions between FoxO, Akt, mTORC1, and C2, as well as those of AMPK and metabolism are shown under dietary restriction and dietary surplus conditions. (For abbreviations see the text.)

We present a model below on the interacting dynamics of the various signal molecules, viz., the Akt, FoxO, AMPK, mTOR complexes (Figure 1) and how the pathways behave under energy/nutrient poor, and energy/nutrient surplus environments (Figure 2).

## The Model

A simplified sequence of events of phosphorylation/dephosphorylation or activation/inactivation cycles of Akt *vis-a-vis* the mTOR complexes can be described as hereunder.

Under energy/nutrient depleted conditions, Akt is in an inhibited state; FoxO and AMPK transcription factors are activated. AMPK activates glycolysis and krebs cycle and generates ATP, but inhibits mTORC1 (Hardie and Hawley, 2001; Liang and Mills, 2013). AMPK also activates autophagy, which recycles the cellular cargo and degrades defective/damaged mitochondria in cells starved of energy (Egan et al., 2011; Hardie, 2011). Within the nucleus, the activation of FoxO leads to transcription of rictor (Chen et al., 2010), IRS (Tsunekawa et al., 2011; Tsuchiya et al., 2012) and enzymes of gluconeogenesis (Zhang et al., 2006; Liu et al., 2011; Shin et al., 2012). Amino acids released from recycled cargo by autophagy release the inhibition on mTORC1 by AMPK and provide the substrates for protein synthesis needed for the reprogramming of metabolism. Activation of mTORC1 also facilitates the translation of transcription products of FoxO like rictor and IRS. The protein rictor promotes the constitution of mTORC2 which then translocates to membrane to activate the Akt S473 phosphorylation (see the text). Since insulin or IGFs are absent under nutrient deprived conditions, it is probable that Akt is activated independent of PI3K/insulin signaling (Brugge et al., 2007; Mahajan and Mahajan, 2012). Activated Akt is liberated from the membrane and migrates into cytoplasm and nucleus. In the cytoplasm it phosphorylates As 160, which leads to translocation of GLUT4 to the membrane. It has been shown that both Akt S473 and mTORC2 are key factors in GLUT4 translocation (Zong et al., 2009; Kumar et al., 2010; Fukuda, 2011; Kim et al., 2011). Within the nucleus, Akt phosphorylates FoxO, which then translocates into the cytoplasm (Guertin et al., 2006). In the cytoplasm, active AMPK drives FoxO into the mitochondria and in cooperation with the SIRTUIN3 activates mitochondrial respiration (Peserico et al., 2013).

This increases production of ATP, raising the levels of ATP/AMP ratio. Enhanced ATP/AMP ratio stabilizes Akt, which targets TSC2, PRAS40; this results in further activation of mTORC1. Coupled with the exclusion of FoxO from the nucleus, activation of mTORC1 leads to the inhibition of rictor and disassembly mTORC2, which reduces the mTORC2 mediated S473 phosphorylation of Akt and release of inhibition on AS 160 and making GLUT4 dysfunctional.

### The nutrient status of organisms appears to play a critical role in this energy and signaling dynamics

Under dietary restriction, limited entry of glucose keeps the rise in ATP:AMP ratio at the bare minimum levels to maintain living processes, mTORC1 remains largely inhibited and IIS is not in the picture (Mercken et al., 2013). When the nutrients are maintained in a balanced state, especially during the growth phase of the organisms, the energy expenditure due to active mTORC1 reduces the ATP/AMP ratio destabilizing Akt and the alternate activation, reactivation cycle of Akt, and glucose entry regulate IIS. Higher activation of mTORC1 due to enriched nutrients/amino acids inside the cells might lead to inhibition of rictor (mTORC2) and a feedback inhibition of IIS (see the text). Inhibition of autophagy/mitophagy by mTORC1 will also produce reaction oxygen species (ROS) and create conditions of inflammation (Thiem et al., 2013). It is possible that when ROS generated during active biosynthetic processes are under control, the protective phase of inflammation might lead to activation of cytokines and TNFα, and promote phosphorylation of AktS473 exclusively even in the absence of mTORC2 (O’Toole et al., 2001). This retains the GLUT4 active at the membrane and promotes the replenishment of glucose in the cell for ATP production. But under malnutrition and surplus nutrients, especially in aging organisms, the deregulation of Akt activation, deactivation cycles promote the stress activated state. Increased levels of cytokines and stress activated kinases like JNK (Salminen and Kaarniranta, 2013) under such situations hampers both IIS and the glucose flux resulting in the state of insulin resistance (Figure 2).

## Conclusion

In summary, we suggest that the nutrient inputs, energy cycles regulate the functions of several signal molecules. Akt is positioned at the central hub of regulating these interactions. The reciprocal relation that exists between the two activating phosphorylation sites, T308 and S473, of Akt and the two mTOR complexes, C1 and C2, forms the central controlling hub that regulates cellular function. The energy charge, the ratio of concentration of ATP to that of AMP, decides the active and inactive state of Akt. In general, the stability of Akt phosphorylations is coupled to high ATP:AMP ratio in cells. It activates mTORC1 and the energy consuming biosynthetic processes. Under nutrient deprived conditions, active FoxO transcribes rictor promoting constitution of mTORC2; phosphorylation of T308 and activation of Akt may be independent of IIS and Akt maybe stable for short time, which is required for maintenance of basal metabolism.

## Conflict of Interest Statement

The authors declare that the research was conducted in the absence of any commercial or financial relationships that could be construed as a potential conflict of interest.

## Abbreviations

AH/PH domain, a unique N-terminal domain of the AKT protein which contains pleckstrin homology domain for binding of PIP3; Akt, protein kinase B (T308, S473 – phosphorylated sites threonine 308 and serine 473); FoxO, fork head transcription factors of O group; GLUT, glucose transporter; GSK3β, glycogen synthase kinase3β; IGF, insulin growth factor; IRS, insulin receptor substrate; mTORC1, 2, mechanistic target of rapamycin complex 1 and 2 (mTOR, formerly known as mammalian target of rapamycin); PDK2, phosphoinositide dependent kinase2 (a putative kinase thought to phosphorylate Akt on S473); PI3K, phosphatidylinositol 3-kinases; PIP2, phosphatidylinositol 4,5-bisphosphate; PIP3, phosphatidylinositol 3,4,5-trisphosphate; PIPDK, phosphoinositide dependent kinase 1 (the abbreviation PIPDK is preferred over the original PDK1 in the article to avoid confusion with the pyruvate dehydrogenase kinase, which is also abbreviated as PDK1 in the literature); rictor, a component of mTORC2; ROS, reaction oxygen species; S6K, the p70 ribosomal S6K; Sestrins, stress response proteins.

## References

[B1] Alessi D. R. Andjelkovic M. Caudwell B. Cron P. Morrice N. Cohen P. (1996). Mechanism of activation of protein kinase B by insulin and IGF-1. EMBO J. 15, 6541–6551 8978681 PMC452479

[B2] Alessi D. R. James S. R. Downes C. P. Holmes A. B. Gaffney P. R. Reese C. B. (1997). Characterization of a 3-phosphoinositide-dependent protein kinase which phosphorylates and activates protein kinase Balpha. Curr. Biol. 7, 261–269 10.1016/S0960-9822(06)00122-9 9094314

[B3] Ananthanarayanan B. Fosbrink M. Rahdar M. Zhang J. (2007). Live-cell molecular analysis of Akt activation reveals roles for activation loop phosphorylation. J. Biol. Chem. 282, 36634–36641 10.1074/jbc.M706227200 17928291

[B4] Atkinson D. E. (1968). The energy charge of the adenylate pool as a regulatory parameter. Interaction with feedback modifiers. Biochemistry 7, 4030–4034 10.1021/bi00851a033 4972613

[B5] Bayascas J. R. Alessi D. R. (2005). Regulation of Akt/PKB Ser473 phosphorylation. Mol. Cell 18, 143–145 10.1016/j.molcel.2005.03.020 15837416

[B6] Bellacosa A. Testa J. R. Staal S. P. Tsichlis P. N. (1991). A retroviral oncogene, akt, encoding a serine-threonine kinase containing an SH2-like region. Science 254, 274–277 10.1126/science.1833819 1833819

[B7] Boosani C. S. Agrawal D. K. (2013). PTEN modulators: a patent review. Expert Opin. Ther. Pat. 23, 569–580 10.1517/13543776.2013.768985 23379765 PMC3653164

[B8] Bozulic L. Surucu B. Hynx D. Hemmings B. A. (2008). PKBalpha/Akt1 acts downstream of DNA-PK in the DNA double-strand break response and promotes survival. Mol. Cell 30, 203–213 10.1016/j.molcel.2008.02.024 18439899

[B9] Breuleux M. Klopfenstein M. Stephan C. Doughty C. A. Barys L. Maira S. M. (2009). Increased AKT S473 phosphorylation after mTORC1 inhibition is rictor dependent and does not predict tumor cell response to PI3K/mTOR inhibition. Mol. Cancer Ther. 8, 742–753 10.1158/1535-7163.MCT-08-0668 19372546 PMC3440776

[B10] Brugge J. Hung M. C. Mills G. B. (2007). A new mutational AKTivation in the PI3K pathway. Cancer Cell 12, 104–107 10.1016/j.ccr.2007.07.014 17692802

[B11] Calleja V. Laguerre M. Larijani B. (2009a). 3-D structure and dynamics of protein kinase B-new mechanism for the allosteric regulation of an AGC kinase. J. Chem. Biol. 2, 11–25 10.1007/s12154-009-0016-8 19568789 PMC2682354

[B12] Calleja V. Laguerre M. Parker P. J. Larijani B. (2009b). Role of a novel PH-kinase domain interface in PKB/Akt regulation: structural mechanism for allosteric inhibition. PLoS Biol. 7:e17 10.1371/journal.pbio.1000017 19166270 PMC2628406

[B13] Chan T. O. Tsichlis P. N. (2001). PDK2: a complex tail in one Akt. Sci. STKE 2001, e1 10.1126/stke.2001.66.pe1 11752635

[B14] Chan T. O. Zhang J. Rodeck U. Pascal J. M. Armen R. S. Spring M. (2011). Resistance of Akt kinases to dephosphorylation through ATP-dependent conformational plasticity. Proc. Natl. Acad. Sci. U.S.A. 108, E1120–E1127 10.1073/pnas.1109879108 22031698 PMC3219155

[B15] Chandrasekher G. Sailaja D. (2004). Phosphatidylinositol 3-kinase (PI-3K)/Akt but not PI-3K/p70 S6 kinase signaling mediates IGF-1-promoted lens epithelial cell survival. Invest. Ophthalmol. Vis. Sci. 45, 3577–3588 10.1167/iovs.04-0279 15452065

[B16] Chen C. C. Jeon S. M. Bhaskar P. T. Nogueira V. Sundararajan D. Tonic I. (2010). FoxOs inhibit mTORC1 and activate Akt by inducing the expression of Sestrin3 and rictor. Dev. Cell 18, 592–604 10.1016/j.devcel.2010.03.008 20412774 PMC3031984

[B17] Coffer P. J. Woodgett J. R. (1991). Molecular cloning and characterisation of a novel putative protein-serine kinase related to the cAMP-dependent and protein kinase C families. Eur. J. Biochem. 201, 475–481 10.1111/j.1432-1033.1991.tb16305.x 1718748

[B18] Dong L. Q. Liu F. (2005). PDK2: the missing piece in the receptor tyrosine kinase signaling pathway puzzle. Am. J. Physiol. Endocrinol. Metab. 289, E187–E196 10.1152/ajpendo.00011.2005 16014356

[B19] Downward J. (1995). Signal transduction. A target for PI(3) kinase. Nature 376, 553–554 7637799 10.1038/376553a0

[B20] Egan D. F. Shackelford D. B. Mihaylova M. M. Gelino S. Kohnz R. A. Mair W. (2011). Phosphorylation of ULK1 (hATG1) by AMP-activated protein kinase connects energy sensing to mitophagy. Science 331, 456–461 10.1126/science.1196371 21205641 PMC3030664

[B21] Eke I. Koch U. Hehlgans S. Sandfort V. Stanchi F. Zips D. (2010). PINCH1 regulates Akt1 activation and enhances radioresistance by inhibiting PP1alpha. J. Clin. Invest. 120, 2516–2527 10.1172/JCI41078 20530873 PMC2898588

[B22] Feng J. Park J. Cron P. Hess D. Hemmings B. A. (2004). Identification of a PKB/Akt hydrophobic motif Ser-473 kinase as DNA-dependent protein kinase. J. Biol. Chem. 279, 41189–41196 10.1074/jbc.M406731200 15262962

[B23] Franke T. F. Yang S. I. Chan T. O. Datta K. Kazlauskas A. Morrison D. K. (1995). The protein kinase encoded by the Akt proto-oncogene is a target of the PDGF-activated phosphatidylinositol 3-kinase. Cell 81, 727–736 10.1016/0092-8674(95)90534-0 7774014

[B24] Frias M. A. Thoreen C. C. Jaffe J. D. Schroder W. Sculley T. Carr S. A. (2006). mSin1 is necessary for Akt/PKB phosphorylation, and its isoforms define three distinct mTORC2s. Curr. Biol. 16, 1865–1870 10.1016/j.cub.2006.08.001 16919458

[B25] Fruman D. A. Meyers R. E. Cantley L. C. (1998). Phosphoinositide kinases. Annu. Rev. Biochem. 67, 481–507 10.1146/annurev.biochem.67.1.481 9759495

[B26] Fukuda M. (2011). TBC proteins: GAPs for mammalian small GTPase Rab? Biosci. Rep. 31, 159–168 10.1042/BSR20100112 21250943

[B27] Gallay N. Dos Santos C. Cuzin L. Bousquet M. Simmonet Gouy V. Chaussade C. (2009). The level of AKT phosphorylation on threonine 308 but not on serine 473 is associated with high-risk cytogenetics and predicts poor overall survival in acute myeloid leukaemia. Leukemia 23, 1029–1038 10.1038/leu.2008.395 19158829

[B28] Gao T. Furnari F. Newton A. C. (2005). PHLPP: a phosphatase that directly dephosphorylates Akt, promotes apoptosis, and suppresses tumor growth. Mol. Cell 18, 13–24 10.1016/j.molcel.2005.03.008 15808505

[B29] Goncharova E. A. Goncharov D. A. Li H. Pimtong W. Lu S. Khavin I. (2011). mTORC2 is required for proliferation and survival of TSC2-null cells. Mol. Cell. Biol. 31, 2484–2498 10.1128/MCB.01061-10 21482669 PMC3133430

[B30] Gu Y. Lindner J. Kumar A. Yuan W. Magnuson M. A. (2011). Rictor/mTORC2 is essential for maintaining a balance between beta-cell proliferation and cell size. Diabetes 60, 827–837 10.2337/db10-1194 21266327 PMC3046843

[B31] Guan H. Song L. Cai J. Huang Y. Wu J. Yuan J. (2011). Sphingosine kinase 1 regulates the Akt/FOXO3a/Bim pathway and contributes to apoptosis resistance in glioma cells. PLoS ONE 6:e19946 10.1371/journal.pone.0019946 21625639 PMC3097221

[B32] Guertin D. A. Stevens D. M. Thoreen C. C. Burds A. A. Kalaany N. Y. Moffat J. (2006). Ablation in mice of the mTORC components raptor, rictor, or mLST8 reveals that mTORC2 is required for signaling to Akt-FOXO and PKCalpha, but not S6K1. Dev. Cell 11, 859–871 10.1016/j.devcel.2006.10.007 17141160

[B33] Guo S. Dunn S. L. White M. F. (2006). The reciprocal stability of FOXO1 and IRS2 creates a regulatory circuit that controls insulin signaling. Mol. Endocrinol. 20, 3389–3399 10.1210/me.2006-0092 16916938

[B34] Hardie D. G. (2011). AMP-activated protein kinase: an energy sensor that regulates all aspects of cell function. Genes Dev. 25, 1895–1908 10.1101/gad.17420111 21937710 PMC3185962

[B35] Hardie D. G. Hawley S. A. (2001). AMP-activated protein kinase: the energy charge hypothesis revisited. Bioessays 23, 1112–1119 10.1002/bies.10009 11746230

[B36] Hill M. M. Andjelkovic M. Brazil D. P. Ferrari S. Fabbro D. Hemmings B. A. (2001). Insulin-stimulated protein kinase B phosphorylation on Ser-473 is independent of its activity and occurs through a staurosporine-insensitive kinase. J. Biol. Chem. 276, 25643–25646 10.1074/jbc.C100174200 11373274

[B37] Hollander M. C. Blumenthal G. M. Dennis P. A. (2011). PTEN loss in the continuum of common cancers, rare syndromes and mouse models. Nat. Rev. Cancer 11, 289–301 10.1038/nrc3037 21430697 PMC6946181

[B38] Humphrey S. J. James D. E. (2012). Uncaging akt. Sci. Signal. 5:e20 10.1126/scisignal.2003085 22569332

[B39] Ikenoue T. Inoki K. Yang Q. Zhou X. Guan K. L. (2008). Essential function of TORC2 in PKC and Akt turn motif phosphorylation, maturation and signalling. EMBO J. 27, 1919–1931 10.1038/emboj.2008.119 18566587 PMC2486275

[B40] Jones P. F. Jakubowicz T. Pitossi F. J. Maurer F. Hemmings B. A. (1991). Molecular cloning and identification of a serine/threonine protein kinase of the second-messenger subfamily. Proc. Natl. Acad. Sci. U.S.A. 88, 4171–4175 10.1073/pnas.88.10.4171 1851997 PMC51620

[B41] Kim J. Kundu M. Viollet B. Guan K. L. (2011). AMPK and mTOR regulate autophagy through direct phosphorylation of Ulk1. Nat. Cell Biol. 13, 132–141 10.1038/ncb2152 21258367 PMC3987946

[B42] King T. D. Song L. Jope R. S. (2006). AMP-activated protein kinase (AMPK) activating agents cause dephosphorylation of Akt and glycogen synthase kinase-3. Biochem. Pharmacol. 71, 1637–1647 10.1016/j.bcp.2006.03.005 16620785 PMC1618797

[B43] Kohn A. D. Kovacina K. S. Roth R. A. (1995). Insulin stimulates the kinase activity of RAC-PK, a pleckstrin homology domain containing ser/thr kinase. EMBO J. 14, 4288–4295 7556070 10.1002/j.1460-2075.1995.tb00103.x PMC394513

[B44] Konishi H. Karakas B. Abukhdeir A. M. Lauring J. Gustin J. P. Garay J. P. (2007). Knock-in of mutant K-ras in nontumorigenic human epithelial cells as a new model for studying K-ras mediated transformation. Cancer Res. 67, 8460–8467 10.1158/0008-5472.CAN-07-0108 17875684

[B45] Kumar A. Lawrence J. C. Jr. Jung D. Y. Ko H. J. Keller S. R. Kim J. K. (2010). Fat cell-specific ablation of rictor in mice impairs insulin-regulated fat cell and whole-body glucose and lipid metabolism. Diabetes 59, 1397–1406 10.2337/db09-1061 20332342 PMC2874700

[B46] Kunkel M. T. Ni Q. Tsien R. Y. Zhang J. Newton A. C. (2005). Spatio-temporal dynamics of protein kinase B/Akt signaling revealed by a genetically encoded fluorescent reporter. J. Biol. Chem. 280, 5581–5587 10.1074/jbc.M411534200 15583002 PMC2913970

[B47] Kwiatkowski D. J. Manning B. D. (2005). Tuberous sclerosis: a GAP at the crossroads of multiple signaling pathways. Hum. Mol. Genet. 14, 251–258 10.1093/hmg/ddi260 16244323

[B48] Laplante M. Sabatini D. M. (2012). mTOR signaling in growth control and disease. Cell 149, 274–293 10.1016/j.cell.2012.03.017 22500797 PMC3331679

[B49] Lee S. E. Sun S. C. Choi H. Y. Uhm S. J. Kim N. H. (2012). mTOR is required for asymmetric division through small GTPases in mouse oocytes. Mol. Reprod. Dev. 79, 356–366 10.1002/mrd.22035 22407942

[B50] Liang J. Mills G. B. (2013). AMPK: a contextual oncogene or tumor suppressor? Cancer Res. 73, 2929–2935 10.1158/0008-5472.CAN-12-3876 23644529 PMC3725287

[B51] Lin K. Lin J. Wu W. I. Ballard J. Lee B. B. Gloor S. L. (2012). An ATP-site on-off switch that restricts phosphatase accessibility of Akt. Sci. Signal. 5, ra37 10.1126/scisignal.2002618 22569334

[B52] Liu H. Fergusson M. M. Wu J. J. Rovira I. I. Liu J. Gavrilova O. (2011). Wnt signaling regulates hepatic metabolism. Sci. Signal. 4, ra6 10.1126/scisignal.2001249 21285411 PMC3147298

[B53] Macreadie I. G. Horaitis O. Verkuylen A. J. Savin K. W. (1991). Improved shuttle vectors for cloning and high-level Cu(2+)-mediated expression of foreign genes in yeast. Gene 104, 107–111 _ANY_ 1916270 10.1016/0378-1119(91)90474-p

[B54] Maehama T. Dixon J. E. (1998). The tumor suppressor. J. Biol. Chem. 273, 13375–13378 10.1074/jbc.273.22.13375 9593664

[B55] Mahajan K. Mahajan N. P. (2012). PI3K-independent AKT activation in cancers: a treasure trove for novel therapeutics. J. Cell. Physiol. 227, 3178–3184 10.1002/jcp.24065 22307544 PMC3358464

[B56] McDonald P. C. Oloumi A. Mills J. Dobreva I. Maidan M. Gray V. (2008). Rictor and integrin-linked kinase interact and regulate Akt phosphorylation and cancer cell survival. Cancer Res. 68, 1618–1624 10.1158/0008-5472.CAN-07-5869 18339839

[B57] Mercken E. M. Crosby S. D. Lamming D. W. Jebailey L. Krzysik-Walker S. Villareal D. (2013). Calorie restriction in humans inhibits the PI3K/AKT pathway and induces a younger transcription profile. Aging Cell.. [Epub ahead of print]. 10.1111/acel.12088 23601134 PMC3714316

[B58] Mester J. Eng C. (2013). When overgrowth bumps into cancer: the PTEN-opathies. Am. J. Med. Genet. C Semin. Med. Genet. 163, 114–121 10.1002/ajmg.c.31364 23613428

[B59] Moore S. F. Hunter R. W. Hers I. (2011). mTORC2-mediated Akt Ser473 phosphorylation is not required for Akt1 activity in human platelets. J. Biol. Chem. 36, 374–387 10.1074/jbc.M110.202341 21592956 PMC3137030

[B60] Nicholson K. M. Anderson N. G. (2002). The protein kinase B/Akt signalling pathway in human malignancy. Cell. Signal. 14, 381–395 10.1016/S0898-6568(01)00271-6 11882383

[B61] Nyakern M. Tazzari P. L. Finelli C. Bosi C. Follo M. Y. Grafone T. (2006). Frequent elevation of Akt kinase phosphorylation in blood marrow and peripheral blood mononuclear cells from high-risk myelodysplastic syndrome patients. Leukemia 20, 230–238 10.1038/sj.leu.2404057 16341040

[B62] Oakhill J. S. Scott J. W. Kemp B. E. (2012). AMPK functions as an adenylate charge-regulated protein kinase. Trends Endocrinol. Metab. 23, 125–132 10.1016/j.tem.2011.12.006 22284532

[B63] Oakhill J. S. Steel R. Chen Z. P. Scott J. W. Ling N. Tam S. (2011). AMPK is a direct adenylate charge-regulated protein kinase. Science 332, 1433–1435 10.1126/science.1200094 21680840

[B64] O’Toole A. Moule S. K. Lockyer P. J. Halestrap A. P. (2001). Tumour necrosis factor-alpha activation of protein kinase B in WEHI-164 cells is accompanied by increased phosphorylation of Ser473, but not Thr308. Biochem. J. 359, 119–127 10.1042/0264-6021:3590119 11563975 PMC1222127

[B65] Peserico A. Chiacchiera F. Grossi V. Matrone A. Latorre D. Simonatto M. (2013). A novel AMPK-dependent FoxO3A-SIRT3 intramitochondrial complex sensing glucose levels. Cell. Mol. Life Sci. 70, 2015–2029 10.1007/s00018-012-1244-6 23283301 PMC11113715

[B66] Ramaiah A. Hathaway J. A. Atkinson D. E. (1964). Adenylate as a metabolic regulator. effect on yeast phosphofructokinase kinetics. J. Biol. Chem. 239, 3619–3622 14257585

[B67] Resjo S. Goransson O. Harndahl L. Zolnierowicz S. Manganiello V. Degerman E. (2002). Protein phosphatase 2A is the main phosphatase involved in the regulation of protein kinase B in rat adipocytes. Cell. Signal. 14, 231–238 10.1016/S0898-6568(01)00238-8 11812651

[B68] Rodrik-Outmezguine V. S. Chandarlapaty S. Pagano N. C. Poulikakos P. I. Scaltriti M. Moskatel E. (2012). mTOR kinase inhibition causes feedback-dependent biphasic regulation of AKT signaling. Cancer Discov. 1, 248–259 10.1158/2159-8290.CD-11-0085 22140653 PMC3227125

[B69] Salminen A. Kaarniranta K. (2013). Insulin/IGF-1 paradox of aging: regulation via AKT/IKK/NF-kappaB signaling. Cell. Signal. 22, 573–577 10.1016/j.cellsig.2009.10.006 19861158

[B70] Sarbassov D. D. Guertin D. A. Ali S. M. Sabatini D. M. (2005). Phosphorylation and regulation of Akt/PKB by the rictor-mTOR complex. Science 307, 1098–1101 10.1126/science.1106148 15718470

[B71] Scheid M. P. Marignani P. A. Woodgett J. R. (2002). Multiple phosphoinositide 3-kinase-dependent steps in activation of protein kinase B. Mol. Cell. Biol. 22, 6247–6260 10.1128/MCB.22.17.6247-6260.2002 12167717 PMC134003

[B72] Schwenk R. W. Dirkx E. Coumans W. A. Bonen A. Klip A. Glatz J. F. (2010). Requirement for distinct vesicle-associated membrane proteins in insulin- and AMP-activated protein kinase (AMPK)-induced translocation of GLUT4 and CD36 in cultured cardiomyocytes. Diabetologia 53, 2209–2219 10.1007/s00125-010-1832-7 20582536 PMC2931635

[B73] Semenza G. L. (2008). Tumor metabolism: cancer cells give and take lactate. J. Clin. Invest. 118, 3835–3837 10.1172/JCI37373 19033652 PMC2582934

[B74] Shin D. J. Joshi P. Hong S. H. Mosure K. Shin D. G. Osborne T. F. (2012). Genome-wide analysis of FoxO1 binding in hepatic chromatin: potential involvement of FoxO1 in linking retinoid signaling to hepatic gluconeogenesis. Nucleic Acids Res. 40, 11499–11509 10.1093/nar/gks932 23066095 PMC3526316

[B75] Stokoe D. Stephens L. R. Copeland T. Gaffney P. R. Reese C. B. Painter G. F. (1997). Dual role of phosphatidylinositol-3,4,5-trisphosphate in the activation of protein kinase B. Science 277, 567–570 10.1126/science.277.5325.567 9228007

[B76] Stronach E. A. Chen M. Maginn E. N. Agarwal R. Mills G. B. Wasan H. (2011). DNA-PK mediates AKT activation and apoptosis inhibition in clinically acquired platinum resistance. Neoplasia 13, 1069–1080 22131882 10.1593/neo.111032 PMC3223610

[B77] Tazzari P. L. Cappellini A. Grafone T. Mantovani I. Ricci F. Billi A. M. (2004). Detection of serine 473 phosphorylated Akt in acute myeloid leukaemia blasts by flow cytometry. Br. J. Haematol. 126, 675–681 10.1111/j.1365-2141.2004.05121.x 15327518

[B78] Thayyullathil F. Chathoth S. Shahin A. Kizhakkayil J. Hago A. Patel M. (2011). Protein phosphatase 1-dependent dephosphorylation of Akt is the prime signaling event in sphingosine-induced apoptosis in Jurkat cells. J. Cell. Biochem. 112, 1138–1153 10.1002/jcb.23033 21308747

[B79] Thiem S. Pierce T. P. Palmieri M. Putoczki T. L. Buchert M. Preaudet A. (2013). mTORC1 inhibition restricts inflammation-associated gastrointestinal tumorigenesis in mice. J. Clin. Invest. 123, 767–781 10.1172/JCI65086 23321674 PMC3561832

[B80] Tsuchiya K. Tanaka J. Shuiqing Y. Welch C. L. DePinho R. A. Tabas I. (2012). FoxOs integrate pleiotropic actions of insulin in vascular endothelium to protect mice from atherosclerosis. Cell Metab. 15, 372–381 10.1016/j.cmet.2012.01.018 22405072 PMC3315846

[B81] Tsunekawa S. Demozay D. Briaud I. McCuaig J. Accili D. Stein R. (2011). FoxO feedback control of basal IRS-2 expression in pancreatic beta-cells is distinct from that in hepatocytes. Diabetes 60, 2883–2891 10.2337/db11-0340 21933986 PMC3198101

[B82] Vadlakonda L. Pasupuleti M. Pallu R. (2013). Role of PI3K-AKT-mTOR and Wnt signaling pathways in transition of G1-S phase of cell cycle in cancer cells. Front. Oncol. 3:85 10.3389/fonc.2013.00085 23596569 PMC3624606

[B83] Vincent E. E. Elder D. J. Thomas E. C. Phillips L. Morgan C. Pawade J. (2011). Akt phosphorylation on Thr308 but not on Ser473 correlates with Akt protein kinase activity in human non-small cell lung cancer. Br. J. Cancer 104, 1755–1761 10.1038/bjc 21505451 PMC3111153

[B84] Weidberg H. Elazar Z. (2011). TBK1 mediates crosstalk between the innate immune response and autophagy. Sci. Signal. 4, e39 10.1126/scisignal.2002355 21868362

[B85] White M. F. (2003). Insulin signaling in health and disease. Science 302, 1710–1711 10.1126/science.1092952 14657487

[B86] Wu N. Zheng B. Shaywitz A. Dagon Y. Tower C. Bellinger G. (2013). AMPK-dependent degradation of TXNIP upon energy stress leads to enhanced glucose uptake via GLUT1. Mol. Cell 49, 1167–1175 10.1016/j.molcel.2013.01.035 23453806 PMC3615143

[B87] Xie X. Zhang D. Zhao B. Lu M. K. You M. Condorelli G. (2011). IkappaB kinase epsilon and TANK-binding kinase 1 activate AKT by direct phosphorylation. Proc. Natl. Acad. Sci. U.S.A. 108, 6474–6479 10.1073/pnas.1016132108 21464307 PMC3081021

[B88] Xu W. Yuan X. Jung Y. J. Yang Y. Basso A. Rosen N. (2003). The heat shock protein 90 inhibitor geldanamycin and the ErbB inhibitor ZD1839 promote rapid PP1 phosphatase-dependent inactivation of AKT in ErbB2 overexpressing breast cancer cells. Cancer Res. 63, 7777–7784 14633703

[B89] Yang J. Cron P. Thompson V. Good V. M. Hess D. Hemmings B. A. (2002). Molecular mechanism for the regulation of protein kinase B/Akt by hydrophobic motif phosphorylation. Mol. Cell 9, 1227–1240 10.1016/S1097-2765(02)00550-6 12086620

[B90] Young A. R. Narita M. Ferreira M. Kirschner K. Sadaie M. Darot J. F. (2009). Autophagy mediates the mitotic senescence transition. Genes Dev. 23, 798–803 10.1101/gad.519709 19279323 PMC2666340

[B91] Zhang H. H. Lipovsky A. I. Dibble C. C. Sahin M. Manning B. D. (2006). S6K1 regulates GSK3 under conditions of mTOR-dependent feedback inhibition of Akt. Mol. Cell 24, 185–197 10.1016/j.molcel.2006.09.019 17052453 PMC1880887

[B92] Zhu Y. Pereira R. O. O’Neill B. T. Riehle C. Ilkun O. Wende A. R. (2013). Cardiac PI3K-Akt impairs insulin-stimulated glucose uptake independent of mTORC1 and GLUT4 translocation. Mol. Endocrinol. 27, 172–184 10.1210/me.2012-1210 23204326 PMC3545208

[B93] Zong H. Bastie C. C. Xu J. Fassler R. Campbell K. P. Kurland I. J. (2009). Insulin resistance in striated muscle-specific integrin receptor beta1-deficient mice. J. Biol. Chem. 284, 4679–4688 10.1074/jbc.M807408200 19064993 PMC2640962

